# [^18^F]FDG whole-body PET-MR including an integrated breast MR protocol for locoregional and distant staging in breast cancer patients—a feasibility study

**DOI:** 10.1186/s13244-024-01830-5

**Published:** 2024-10-09

**Authors:** Thiemo J. A. van Nijnatten, Cornelis M. de Mooij, Cristina Mitea, Janneke Houwers, Maaike de Boer, Marjolein L. Smidt, Felix M. Mottaghy, Joachim E. Wildberger

**Affiliations:** 1 Department of Radiology and Nuclear Medicine, Maastricht University Medical Center+, Maastricht, The Netherlands; 2 GROW—Research Institute for Oncology and Reproduction, Maastricht University Medical Center+, Maastricht, The Netherlands; 3 Division of Internal Medicine, Department of Medical Oncology, Maastricht University Medical Center+, Maastricht, The Netherlands; 4 Department of Surgery, Maastricht University Medical Center+, Maastricht, The Netherlands; 5 Department of Nuclear Medicine, University Hospital, RWTH Aachen University, Aachen, Germany

**Keywords:** Breast neoplasms, Positron emission tomography, Computed tomography, Magnetic resonance imaging, Neoplasm staging

## Abstract

**Purpose:**

To investigate in a feasibility study the combination of [^18^F]FDG whole-body (WB) positron emission tomography-magnetic resonance (PET-MR), including an integrated breast MR within a single protocol for locoregional and distant staging in breast cancer patients.

**Methods:**

Consecutive patients with breast cancer diagnoses according to conventional imaging modalities (full-field digital mammography (FFDM) and ultrasound (US)) were prospectively included. All patients underwent [^18^F]FDG WB PET-MR, including an integrated dedicated breast MR (prone position) and WB PET-MR (supine position) protocol. Results of [^18^F]FDG WB PET-MR, including integrated breast MR, versus conventional imaging modalities were compared.

**Results:**

From April 2021–April 2022, 28 patients were included. On conventional imaging, cT1-2 breast cancer was present in 22 (FFDM) and 23 (US) out of 28 patients. With regard to clinical nodal status, eight patients were considered cN0, eighteen cN1 (1-3 suspicious lymph nodes), and two patients were cN2 (four suspicious axillary lymph nodes/internal mammary lymph node metastasis). [^18^F]FDG WB PET-MR, including an integrated breast MR protocol, upstaged clinical tumor status in two patients and clinical nodal status in nine patients according to both [^18^F]FDG WB PET-MR and breast MR findings. In addition, distant metastases were detected in three patients (liver/bone), and another patient was diagnosed with a synchronous primary tumor (lung cancer).

**Conclusion:**

[^18^F]FDG WB PET-MR, including an integrated breast MR within a single protocol in breast cancer patients, is feasible and provides a promising new approach in breast cancer patients with regard to locoregional and distant staging.

**Critical relevance statement:**

[^18^F]FDG whole-body PET-MR, including an integrated breast MR protocol, is feasible and allows locoregional and distant staging within a single imaging exam in breast cancer patients.

**Key Points:**

- [^18^F]FDG PET-MR allows the combination of breast MR and whole-body staging.
- Therefore, a single protocol of whole-body [^18^F]FDG PET-MR, including an integrated breast MRI, is investigated.
- [^18^F]FDG PET-MR, including an integrated breast MR is feasible and can be considered in daily clinical practice.

**Graphical Abstract:**

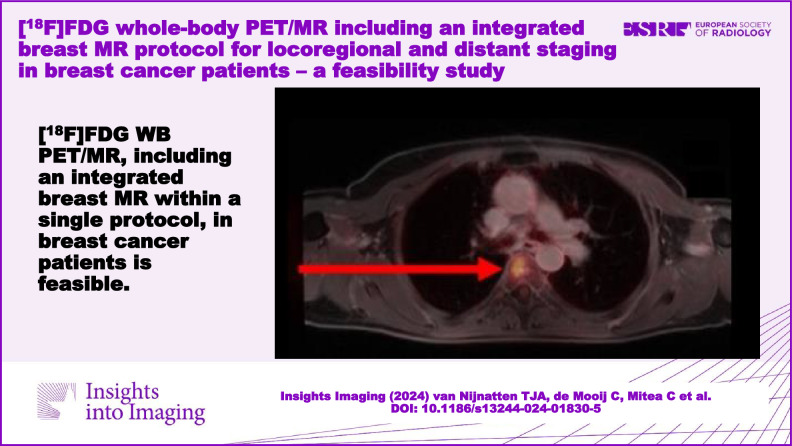

## Introduction

Breast cancer is the most common type of invasive cancer among women worldwide [1]. The mean 5-year overall survival rate after breast cancer diagnosis is currently 90.6%, and varies depending on breast cancer stage: 99.1% in localized breast cancer, 86.1% in the presence of regional lymph node metastases, and 30.0% in the presence of distant metastases [2].

A breast cancer diagnosis can be determined with conventional imaging modalities, such as full-field digital mammography (FFDM) and ultrasound (US) of the breast and axilla complemented with pathology [3]. Next, additional imaging techniques, such as breast magnetic resonance (MR), can be considered to improve locoregional staging, for instance, in the case of preoperative staging, to evaluate the extent of disease or for baseline and response monitoring of patients treated with neoadjuvant systemic therapy [4].

According to the current guidelines, 18F-fluorodeoxyglucose ([^18^]FDG) positron emission tomography (PET) imaging can be considered to rule out distant metastases in recently diagnosed breast cancer patients, for instance, because of large tumor size, regional lymph node metastases, or recurrent breast cancer [5–7]. Consequently, a subgroup of recently diagnosed breast cancer patients requires two additional imaging modalities in terms of breast MR and [^18^F]FDG PET/CT. Yet, since the introduction of [^18^F]FDG PET-MR as opposed to [^18^F]FDG PET/CT, a combination of both techniques (i.e., breast MR and [^18^F]FDG whole-body (WB) PET-MR) in locoregional and distant staging can serve as an advantageous alternative with previously reported non-inferior diagnostic performance [8]. Therefore, we propose a combined imaging protocol by using [^18^F]FDG WB PET-MR including an integrated breast MR protocol. Consequently, patients will only require a ‘one-stop shop’ approach for locoregional and distant staging within one imaging exam. The current feasibility study investigates the potential of such a protocol. In addition, diagnostic performance in comparison to conventional imaging modalities (i.e., FFDM and US) will be analyzed.

## Methods

### Patient selection

From April 2021 until April 2022, all consecutive patients with a recent primary breast cancer diagnosis or patients with a recurrent breast cancer diagnosis requiring further imaging evaluation as part of their diagnostic work-up according to the current national breast cancer guideline, in terms of locoregional (breast MR) and distant staging ([^18^F]FDG WB PET-MR), were included in the current study. According to the Dutch breast cancer guideline in 2018, distant staging with [^18^F]FDG examination can be considered in recently diagnosed breast cancer patients with tumor size larger than 50 mm (i.e., clinical tumor status (cT) 3–4), presence of axillary lymph node metastasis (i.e., clinical nodal status (cN) 1–3) with the potential of neoadjuvant systemic therapy or recurrent breast cancer [9].

Breast cancer diagnosis was confirmed with core-needle biopsy, to obtain histopathologic information including receptor status (estrogen (ER), progesterone (PR) and Human Epidermal growth factor Receptor 2 (HER2)). In the case of suspicious axillary lymph node findings, the core-needle biopsy was performed to confirm axillary lymph node metastases. In the case of suspicious distant metastases on imaging findings, the biopsy was performed to confirm distant metastases of at least one suspicious lesion.

Medical ethical approval was obtained for this single-center feasibility study (METC 2022-3121). The necessity to acquire informed consent from study subjects was waived by the local medical ethics committee.

### Conventional imaging

Conventional imaging consisted of FFDM performed on a Senographe Essential (GE Healthcare, Chalfont St Giles, UK). In addition, breast and axillary ultrasound (US) was performed on an RS80A ultrasound system (Samsung Medison Co., Ltd., Seoul, South Korea) with a 3–12 MHz linear array transducer.

### Imaging protocol: [^18^F]FDG WB PET-MR including integrated breast MR

Before administration of the tracer 3 MBq/kg bodyweight [^18^F]FDG, patients fasted for at least four hours. Blood glucose levels were verified to be lower than 11 mmol/L. All exams were performed on a 3.0-T PET-MR system (Biograph mMR; Siemens Healthineers, Erlangen, Germany).

After a resting period of 30 min after administration of [^18^F]FDG, patients were placed on the PET-MR system in a prone position with elevated arms, using a 16-channel breast coil (Rapid Biomedical, Rimpar, Germany) to perform the breast MR. The breast MR protocol consisted of a two-dimensional T2W turbo spin-echo sequence without fat suppression, diffusion-weighted imaging (DWI) with fat suppression (*B*-values 50, 150 and 800, respectively) and, after injection of the gadolinium-based contrast agent Gadobutrol (Gadovist^®^, Bayer Health Care, Berlin, Germany), a dynamic contrast-enhanced (DCE)-T1W sequence with fat suppression, in accordance with the reported breast MR protocol preferences of the European Society of Breast Imaging [4] (Table 1).

**Table 1:** Overview of breast MR and ([^18^F]FDG) WB PET-MR protocols

Breast MR	[^18^F]FDG WB PET-MR
T2W turbo spin-echo FOV: 340 mm, voxel size: 0.9 × 0.8 × 3.0 mm, TR: 6410 ms, TE: 83 ms, acquisition time: 5 min 28 s, turbo factor 11, flip angle: 80°	Contrast-enhanced T1W FOV: 480 mm, voxel size: 0.6 × 0.6 × 3.0 mm, TR: 4.50 ms, TE 1.26 ms, flip angle: 9°
DWI (*B*-values 50, 150, 800) FOV: 340 mm, voxel size: 1.2 × 1.2 × 5.0 mm, acquisition time: 4 min 21 s	T2W fast spin-echo FOV 450 mm, voxel size: 1.4 × 1.4 × 5.5 mm, TR 1500 ms, TE 113 ms, flip angle: 90°
DCE-T1W FOV 340 mm, voxel size: 0.9 × 0.9 × 1.2 mm, TR: 4.77 ms, TE: 1.76 ms, acquisition time: 9 min 2 s, flip angle: 10°	DWI liver region (*B*-values 50, 800, 1400) FOV: 430 mm, voxel size: 1.7 × 1.7 × 5.0 mm, TR 8680 ms, TE 52 ms

*WB* whole-body, *T2W* T2 weighted, *DWI* diffusion-weighted imaging, *DCE* dynamic contrast-enhanced, *T1W* T1 weighted, *FOV* field of view, *TR* repetition time, *TE* echo time, *mm* millimeter, *ms* milliseconds

Approximately 55 min after administration of [^18^F]FDG, patients were switched from a prone position to a supine position in order to perform the [^18^F]FDG WB PET-MR. The protocol consisted of WB fat-suppressed contrast-enhanced T1W sequence, WB T2W sequence, and DWI of the liver region (*B*-values 50, 800 and 1400, respectively) (Table 1), by using the primary WB coil of the PET/MR system.

Three-dimensional iterative reconstruction was performed for the PET images of the PET-MR system, with automatic attenuation correction by implementation of a four-compartment model attenuation map (Dixon-based μ-map).

### Image evaluation

All breast MR exams were evaluated according to the fifth edition of the BI-RADS lexicon in consensus by two readers, a final-year resident in breast imaging (T.v.N.) and a breast radiologist (J.H.) with more than 15 years of breast imaging experience, by using software on a Sectra Workstation IDS7 (version 23.1.10, Sectra Group, Linköping, Sweden). The total number of suspicious regional lymph nodes on breast MR was based on previously described criteria, including irregular margins, inhomogeneous cortex, perifocal edema, absence of the fatty hilum, asymmetric lymph nodes as opposed to the contralateral side, and absence of chemical shirt artefacts [10].

All [^18^F]FDG WB PET-MR exams were evaluated according to the recommendations of the Society of Nuclear Medicine and Molecular Imaging for oncological [^18^F]FDG PET/CT and recent consensus recommendations on [^18^F]FDG WB PET-MR for oncology in consensus by two readers, a final-year resident in nuclear medicine (T.v.N.) and a nuclear medicine physician (C.M.) with more than 10 years of experience in nuclear medicine, by using a dedicated post-processing environment including state-of-the-art software (Syngo.via 6.4 as software, Siemens Healthcare, Erlangen, Germany) [11, 12]. The total number of suspicious regional lymph nodes on [^18^F]FDG WB PET-MR was based on the number of hypermetabolic lymph nodes.

### Statistical analysis

Findings on [^18^F]FDG WB PET-MR, including integrated breast MR, were compared to conventional imaging findings (i.e., FFDM and US) in terms of clinical tumor size (cT), clinical nodal status (cN) and distant status (cM), according to the 8^th^ edition of TNM classification. Descriptive statistics were performed using SPSS software (version 27, IBM Corp., Armonk, NY, USA).

## Results

### General characteristics

A total of 28 women with recently diagnosed breast cancer underwent [^18^F]FDG WB PET-MR, including an integrated breast MR. Four patients had recurrent breast cancer, and 24 were primary diagnoses of breast cancer. The mean age was 60.2 years. Histology of the breast cancer was considered invasive carcinoma of no special type (NST) in twenty-five patients. Two patients had invasive lobular cancer, and one patient had mucinous cancer (Table 2).

**Table 2:** General characteristics

Number of patients	28
Mean age (years) (range)	60.2 (32–82)
Breast cancer diagnosis (%)	
Primary	24 (85.7)
Recurrent	4 (14.3)
Histology (%)	
Invasive carcinoma NST	25 (89.3)
Invasive lobular cancer	2 (7.1)
Mucinous cancer	1 (3.6)
Receptor status (%)	
ER/PR + HER2−	13 (46.4)
ER/PR + HER2+	8 (28.6)
ER/PR-HER2+	1 (3.6)
Triple negative	6 (21.4)

*NST* no special type, *ER* estrogen, *PR* progesterone, *HER2* human epidermal growth factor receptor 2

### Imaging findings

**Fig. 1:**
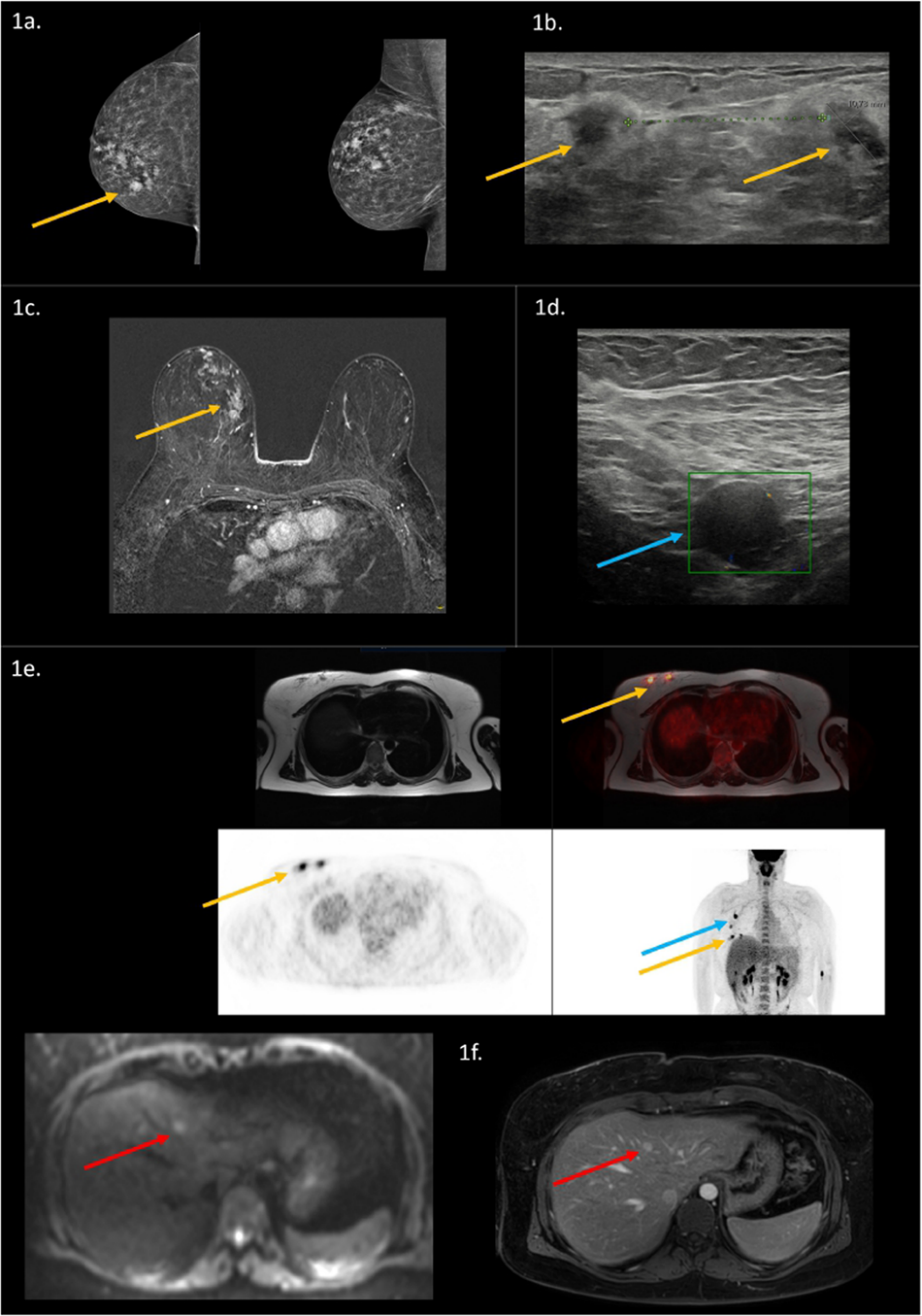
Example of a 40-year-old woman with a palpable lump in her right breast. FFDM demonstrated multifocal masses (orange arrow) in the upper inner quadrant of the right breast, the largest mass diameter was 2.2 cm (**a**). Histopathology confirmed a triple negative breast cancer, invasive carcinoma NST. US confirmed multiple hypo-echoic suspicious masses (orange arrow), the largest mass diameter was 1.1 cm (**b**). Two suspicious axillary lymph nodes (blue arrow) were observed with US (**c**). Breast MR demonstrated a 6.8 cm large enhancing mass with spiculations (orange arrow), diffusion restriction and malignant enhancement pattern (type III) (**d**). Two suspicious axillary lymph nodes were observed on breast MR. [^18^F]FDG WB PET-MR confirmed a hypermetabolic multifocal primary tumor within the right breast (orange arrow), with two hypermetabolic ipsilateral axillary lymph nodes (blue arrow) (**e**). In addition, multiple lesions in the liver were observed (red arrow). The DWI sequence (b800) and T1W sequence after contrast confirmed liver metastases (red arrow) (**f**)

**Fig. 2:**
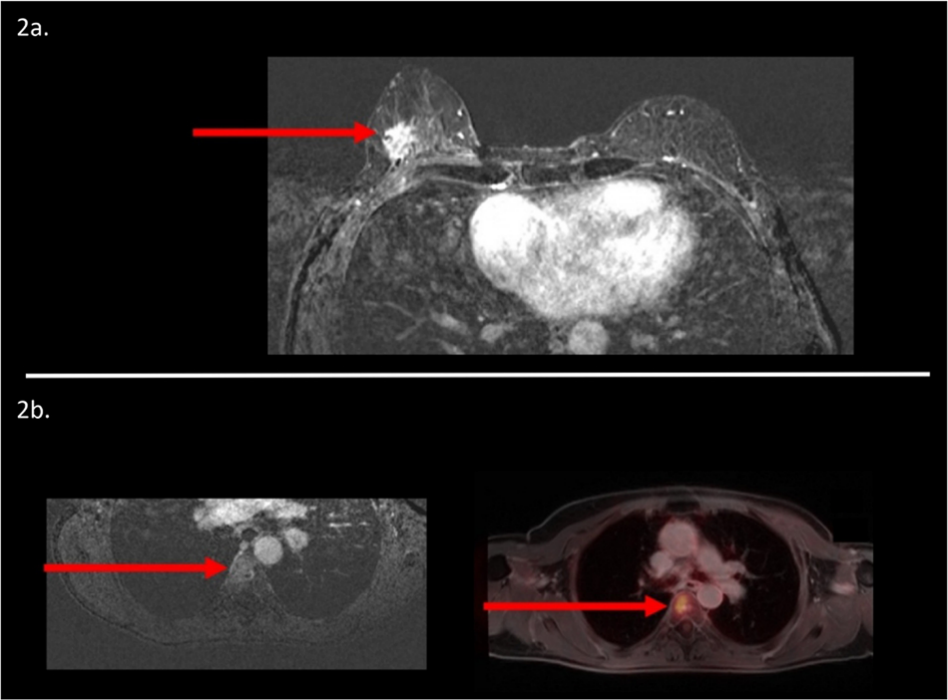
Example of a 58-year-old female patient with a previous history of breast cancer in her left breast. She presented with a palpable lump in her right breast, corresponding with a histopathologically confirmed invasive carcinoma NST in her right breast (diameter 2.2 cm on breast MR, **a**). Fused WB post-contrast T1W sequence with [^18^F]FDG PET revealed a hypermetabolic enhancing lesion in the fourth thoracic vertebra, which was observed on breast MR as well (**b**)

The mean diameter of the primary tumor differed among the imaging modalities, being smallest in the US (25.9 mm) and largest in breast MR (39.8 mm). Regarding the number of suspicious lymph nodes, US reported the lowest mean number of suspicious lymph nodes (1.8 nodes), following [^18^F]FDG WB PET-MR (2.3 nodes), and breast MR (3.0 nodes). In addition, internal mammary lymph nodes were most frequently observed on breast MR (*n* = 6), versus [^18^F]FDG WB PET-MR (*n* = 2) and US (*n* = 1). Distant metastases were most frequently observed on [^18^F]FDG WB PET-MR (*n* = 4) (Fig. 1), from which breast MR already detected three out of four cases with metastatic disease (Fig. 2). Distant metastatic disease was present in two patients with recurrent breast cancer and two patients with primary breast cancer. Further details are described in Table 3.

**Table 3:** Imaging findings conventional imaging versus [^18^F]FDG WB PET-MR (including integrated breast MR)

	Conventional imaging		[^18^F]FDG whole-body PET-MR including integrated breast MR	
	FFDM	US	Breast MR	[^18^F]-FDG WB PET-MR
Mean maximum diameter primary tumor (mm) (range)	31.2 (9–83)	25.9 (7–83)	39.8 (16–86)	-
cT status (%)				
cT1-2	22 (84.6)	23 (95.8)	21 (77.8)	-
cT3-4	4 (14.4)	1 (4.2)	6 (22.2)	
Missing	2	4	1	
Suspicious regional lymph node locations (%)				
Axilla		20 (71.4)	21 (75.0)	17 (60.7)
Internal mammary	-	1 (3.6)	6 (22.2)	2 (7.1)
Periclavicular		1 (3.6)	0	1 (3.6)
Mean total number of suspicious regional lymph nodes (range)	-	1.8 (0–14)	3.0 (0–9)	2.3 (0–7)
cN status (%)				
cN0	-	8 (28.6)	7 (25.0)	11 (29.3)
cN1-2		18 (64.3)	15 (53.6)	15 (53.6)
cN3		2 (7.1)	6 (21.4)	2 (7.1)
Presence of distant metastatic findings (%)	-	-	3 (10.7)	4 (14.3)
Metastatic sites (%)				
Bone	-	-	2	2
Liver			0	1
Lung			1	1
cM (%)				
cM0	-	-	25	24
cM1			3	4

*FFDM* full-field digital mammography, *US* ultrasound, *MR* magnetic resonance imaging, *WB* whole-body, *PET* positron emission tomography, *cT* clinical tumor status, *cN* clinical nodal status, *cM* clinical metastatic status

## Discussion

This is the first feasibility study demonstrating the potential of using a combined imaging protocol consisting of [^18^F]FDG WB PET-MR including an integrated breast MRI; this allows locoregional and distant staging within a single imaging exam in breast cancer patients. These data underline the feasibility of such a combination protocol for clinical use.

According to the current European guidelines, breast MR for locoregional staging can be considered for several reasons, including lobular cancer, multifocality, multicentricity, large discrepancies between conventional imaging and clinical examination, or before neoadjuvant systemic therapy [4, 13]. [^18^F]FDG PET/CT can be considered in the case of large tumor size (≥ cT3), the presence of lymph node metastases at the time of diagnosis with the potential for neoadjuvant systemic therapy, or recurrent breast cancer [5–7]. A previous systematic review demonstrated a non-inferior diagnostic performance of [^18^F]FDG WB PET-MR when compared to [^18^F]FDG (whole-body) PET/CT in breast cancer patients [8]. Consequently, an imaging protocol of [^18^F]FDG WB PET-MR, including an integrated breast MR protocol, allows the performance of two optimal imaging methods within a single protocol. This enhances patient comfort and improves efficiency for the clinical workflow, necessitating only a single appointment to achieve a complete diagnostic work-up prior to the start of treatment.

The total number of suspicious lymph nodes was considered higher on breast MR and [^18^F]FDG WB PET-MR when compared to US findings. This is in line with a study from Goorts et al reporting [^18^F]FDG PET-MR findings in breast cancer patients, resulting in a change in treatment plan by, for instance, extended radiation therapy field when compared to conventional imaging findings [14]. In addition, previous studies reported a higher number of suspicious lymph nodes on [^18^F]FDG PET-MR when compared to [^18^F]FDG PET/CT in breast cancer patients [8, 15]. The difference between a number of suspicious lymph nodes on breast MR and [^18^F]FDG WB PET-MR in the current study can be explained because of differences between both techniques with regard to lymph node metastasis detection: PET suffers from decreased spatial resolution when compared to MRI, while MRI can overestimate the total number of suspicious lymph nodes [16, 17].

A previous study from Biondetti et al reported a non-inferior diagnostic performance for pulmonary nodule detection on [^18^F]FDG WB PET-MR when compared to [^18^F]FDG PET/CT in patients with primary abdominal malignancy, with an overall sensitivity of only 12.1% lung nodule detection on [^18^F]FDG WB PET-MR [18]. Obviously, the sensitivity was strongly depending on pulmonary nodule size: 6% at size 3 mm, 10% at size 4 mm, and 71% at size larger than 7 mm. However, the clinical relevance of these undetected small pulmonary nodules might be limited, since only 1 out of 51 patients in a cohort consisting of known malignancies required upstaging from tumor stage I to IV because of a missed pulmonary nodule on [^18^F]FDG PET-MR [19]. Therefore, the true added value of chest CT over chest MR might be of limited clinical value in patients requiring WB staging with [^18^F]FDG as a PET tracer.

The additional identification of distant metastatic tumor sites in the current study on [^18^F]FDG WB PET-MR versus breast MR was only limited to a single patient (with a total of four patients with distant metastases). However, metastatic tumor sites on breast MR can be subtle, and therefore the additional information of [^18^F]FDG WB PET-MR adds important confidence to discriminate between benign and malignant findings (Fig. 2). Next, the percentage of cT1-2 patients in the current study was relatively high with only a limited amount of suspicious lymph nodes, indicating a relatively low distant metastasis rate on [^18^F]FDG WB PET-MR [20].

This study was limited by the single-center single-vendor study design with only 28 patients. It is, however, expected that implementation of the currently proposed imaging protocol can be rather easily installed on different PET-MR vendor systems as well since two previously existing protocols (breast MR and [^18^F]FDG WB PET-MR, respectively) were combined. It also potentially allows the implementation of this strategy in other cancer types. Of note, this protocol did not include a WB DWI protocol, which might affect the detectability of non-FDG avid abnormalities on [^18^F]FDG WB PET-MR [8]. Next, due to the limited sample size of the current study, there was no subgroup analysis performed. Subgroup analysis would allow stratification by tumor histology and receptor status, both being considered relevant factors for [^18^F]FDG PET interpretation [21]. Next, there was no comparison with the post-surgical treatment specimen due to the increased use of neoadjuvant systemic therapy regimen in recent years, and therefore a true per-patient comparison cannot be performed because of potential treatment effects. Consequently, the comparison between imaging findings and biopsy was performed, rather than a per-patient comparison with the gold standard of post-surgical treatment specimens. Finally, this study did not investigate the financial aspects of the proposed imaging method of [^18^F]FDG WB PET-MR in breast cancer staging, in which the limited global availability of such a system should be kept in mind.

Future studies should investigate an [^18^F]FDG WB PET-MR, including an integrated breast MR protocol in a multivendor multicenter design to demonstrate the added value for clinical use. In addition, patient preference should be taken into account, whether a patient prefers one single examination versus two separate appointments.

To conclude, a combined imaging protocol consisting of [^18^F]FDG WB PET-MR, including an integrated breast MR protocol, is feasible and provides a promising new approach for clinical use in breast cancer patients.

## Data Availability

The dataset generated and analyzed during the study is available from the corresponding author upon reasonable request.

## Abbreviations

[^18^F]FDG: 2-Deoxy-2-fluorine-18 fluoro-d-glucose
CT: Computed tomography
DWI: Diffusion weighted imaging
FFDM: Full-field digital mammography
MRI: Magnetic resonance imaging
PET: Positron emission tomography
US: Ultrasound
WB: Whole-body
